# Rapid Dissolving-Debonding Strategy for Optically Transparent Paper Production

**DOI:** 10.1038/srep17703

**Published:** 2015-12-11

**Authors:** Jinbo Chen, Xiaogang Han, Zhiqiang Fang, Fan Cheng, Bin Zhao, Pengbo Lu, Jun Li, Jiaqi Dai, Steven Lacey, Raphael Elspas, Yuhao Jiang, Detao Liu, Liangbing Hu

**Affiliations:** 1 State Key Laboratory of Pulp and Paper Engineering, South China University of Technology, Guangzhou, Guangdong 510640, People’s Republic of China; 2 Department of Materials Science and Engineering, University of Maryland, College Park, Maryland 20742, United States

## Abstract

Transparent paper is an alternative substrate for electronic devices due to its unique properties. However, energy-intensive and/or time-consuming procedures currently limit the scalable production of transparent paper. In this report, we demonstrate a rapid process to fabricate optically transparent paper with regenerative cellulose fibers (RCFs) by employing a dissolving-debonding strategy. The RCFs have an average width of 19.3 μm and length of several hundred microns and are prepared into transparent paper by vacuum filtration. This new dissolving-debonding approach enables high production efficiency while creating transparent paper with excellent optical and mechanical properties.

Optically transparent paper, composed of microsized wood fibers and/or cellulose nanofibers, has garnered tremendous attention in the past few years as an alternative substrate to glass and plastics for use in electronic devices such as solar cells, transistors, organic light-emitting diodes, touch screens, and antennas^1^,^2^,^3^,^4^,^5^,^6^. Transparent paper possesses quintessential properties such as excellent optical transmittance, strong tensile strength, low surface roughness, and superior thermal stability, which explains its potential to replace conventional substrates. In turn, efficient and environmentally friendly large-scale manufacturing processes are of significance for next generation “green” electronics in terms of production cost and sustainability. Vacuum filtration and casting approaches have been reported to prepare optically transparent nanopaper, however, they rely on time-consuming and energy-intensive nanofiber disintegration steps that utilize a low suspension concentration of cellulose nanofibers and are limited by the fiber’s nanoscale dimensions^7^,^8^,^9^,^10^,^11^,^12^. These factors severely impede the scalable and low-cost manufacturing of transparent nanopaper. Casting or extruding methods are commonly used to fabricate transparent regenerative cellulose films on a large scale^13^,^14^,^15^,^16^, but the solvents used such as N-methylmorpholine N-oxide (NMMO)^17^ and N, N-dimethylacetamide/lithium chloride (DMAc/LiCl)^18^ pose environmental problems as well as reduce production efficiency due to excessive solvent removal steps. The transparent paper made of microsized cellulose fibers demonstrates an optical transmittance of ~90% and a much higher dewatering efficiency than nanopaper, however the process takes 1 hour to produce paper with such high transparency which is not realistic for mass production^4^.

In this report, optically transparent paper is formed from microsized regenerative cellulose fibers (RCFs) through a rapid dissolving and debonding strategy. First, the hydrogen bonds between the cellulose chains breakdown as the raw cellulose fibers are fully dissolved in the ionic liquids (ILs) (dissolving process). Then the obtained cellulose solution is added dropwise into the regenerative solution and vigorously stirred to produce microsized RCFs (debonding process). By adjusting the regenerative solution parameters, microsized RCFs with different fiber dimensions and morphologies are harvested and enabled swift fabrication of optically transparent paper through traditional papermaking processes^19^. Transparent paper made of RCFs has the following advantages over previously reported transparent paper: (1) an economical and efficient filtration method can be applied for mass production of our transparent paper without extreme sacrifices in optical properties and mechanical strength; (2) it takes less time to prepare microscopic RCFs (20 min) and our transparent paper (10 min) with a thickness of 60 μm. This dissolving-debonding strategy sheds light on rapid, low cost production of transparent paper by roll-to-roll manufacturing processes.

## Results

**Figure 1:**
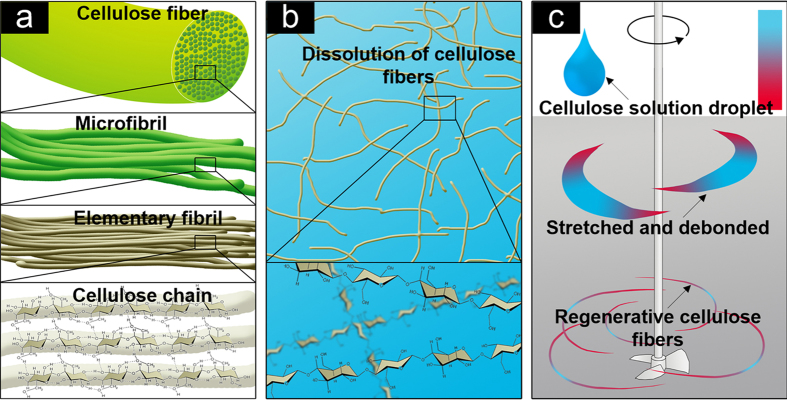
Schematic depicting the dissolving-debonding strategy for RCF preparation. (**a**) The hierarchical structure of cellulose fibers. (**b**) Complete dissolution of the cellulose fibers in ILs by breaking the hydrogen bonds between the cellulose chains. (**c**) The debonding process of the RCFs in the regenerative solution (shown in grey) by shearing forces.

Natural cellulose fibers have a three-dimensional (3D) hierarchical structure which allows cellulose-based materials with dimensions ranging from the nanoscale to the microscale^20^. As shown in Fig. 1a, cellulose chains self-assemble into elementary fibrils with a diameter of 3.5–5 nm and are further bundled into microfibrils. The microfibrils consist of the cell wall of the cellulose fibers. The cellulose fibers were initially added into the ILs with a temperature of 85 °C to prepare the cellulose solution. The hydrogen bonds between the cellulose chains gradually breakdown due to the anion and cation attack from the ILs and the entire dissolving process takes approximately 16 min. When the cellulose fibers are immersed in the anhydrous IL solution, the ILs penetrate through the entire cell wall of the fiber via voids through the inner or outer portions of the cell lumen. The ILs prefer to penetrate into the amorphous sections since the structure is relatively loose compared to the crystalline sections. The original hydrogen bonds between the cellulose chains in the amorphous section are initially broken by the ILs, which converts the solid amorphous sections into liquid cellulose. Subsequently, the hydrogen bonds between the cellulose chains in the crystalline sections are gradually broken by the ILs, which also converts the solid amorphous sections into liquid cellulose. The cellulose fibers are completely dissolved after nearly all the hydrogen bonds between the cellulose chains are broken. The dissolving process was complete after all the hydrogen bonds between the cellulose chains were broken and thus, a homogeneous cellulose solution was achieved. Figure 1b demonstrates that the cellulose chains are well dispersed in the ILs. Afterwards, a debonding procedure was utilized to prepare the microsized RCFs and enable rapid transparent paper production with desirable optical and mechanical properties. The obtained cellulose solution from the dissolving process was slowly dropped into the regenerative solution and stirred at a high speed. The water molecules are instantly attacked causing the hydrogen bond network between the cellulose chains to break once again. A new hydrogen bond network is instituted due to the interactions with the ILs.

**Figure 2:**
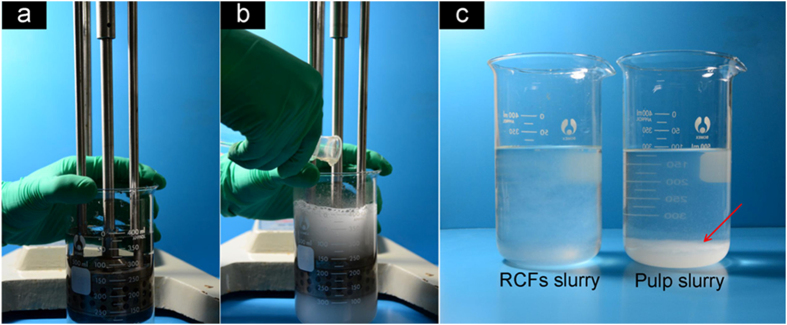
RCF preparation. (a) An emulsifier equipment used to prepare the RCFs. (**b**) The dissolved cellulose solution is added to the regenerative solution to initiate the debonding process. (**c**) Comparative images of the RCFs slurry and pulp slurry in DI water diluents.

In this process, the original cellulose solution droplets were stretched and warped nonlinearly into regenerative fibrous aggregates in a high shearing field (Fig. 1c). Figure 2a,b illustrates the RCF preparation steps where an emulsifier is first added to the regenerative solution followed by the dissolved cellulose solution to initiate the debonding process. The resulting RCFs slurry shows excellent dispersibility with relatively few fiber deposits at the bottom of the deionized (DI) water solution while the typical pulp slurry leads to significant fiber deposition (Fig. 2c). This result indicated that the RCFs have better adaptability in the papermaking process than the pulp slurry even though both fibers have nearly identical microsized dimensions.

A KajaaniFS300 Fiber Analyzer was used to quantitatively analyze the dimensions of the RCFs regenerated in the DI water solution. Table 1 summarizes the fiber dimensions for both raw wood fibers and RCFs. The RCFs have an average fiber length of 0.4 mm and an average fiber width of 19.3 μm, which is much larger than cellulose nanofibers (5–30 nm) and slightly smaller than pulp fibers (Table 1).

**Figure 3:**
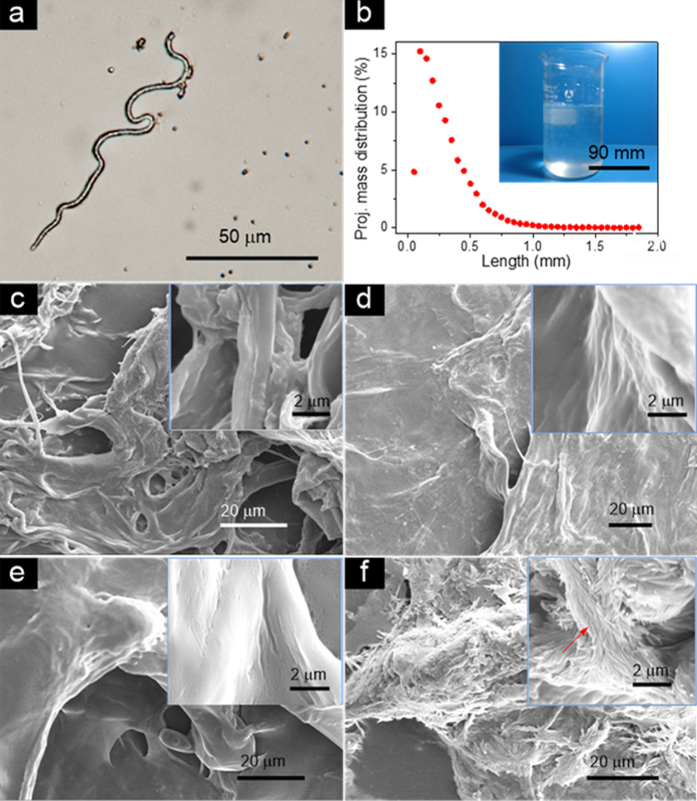
(**a**) Microscopic image of the RCFs. (**b**) Length distribution of the RCFs. The inset in the top right corner is the RCFs suspension. SEM images of the RCFs prepared by different regenerative solutions: (c) 4.5 wt% H_2_SO_4_, (**d**) DI water, (**e**) 4.5 wt% (NH_4_)_2_SO_4_, and (**f**) 2.0 wt% NaOH. (**c–f**) The insets in the top right corner are magnified SEM images of the RCFs.

The curl and kink index of the RCFs is recorded and saved automatically by the KajaaniFS300 Fiber Analyzer. The curl index refers to the gradual and continuous curvature of the fiber^21^,^22^, while the Kink index is indicative of the abrupt change in the fiber’s curvature^21^,^23^. The curl index and kink index of the raw pulp fibers and RCFs are described using Equations (1) and (2), respectively. RCFs have a much higher curl index (52.2%) but a lower kink index (10.9 1/m) compared to pulp fibers, which explains the curvy and weavable nature of the RCFs. The fine content of the RCFs (fiber length less than 200 μm, 47.2%) is much higher than that of regular wood fibers (8.3%). This demonstrates that nearly half of the RCFs have a fiber length less than 200 μm (Fig. 3b). However, the basic hydroxyl groups on the surface of the RCFs enable them to bond tightly through hydrogen bonding pathways, which is similar to regular paper and nanopaper.


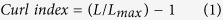



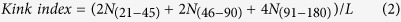


where *L* is the fiber contour length and *L*_*max*_ is the actual fiber length which follows the fiber’s curvature and is therefore the longest dimension. In Equation (2), *N* is the number of kinks in a specific angular range (i.e. the subscript of N is the kink angle range) within the total sample and *L* is the total fiber length.

Optical microscopy was used to observe the morphology of the RCFs from the DI water solution (Fig. 3a). The RCFs slurry is shown in the inset of Fig. 3b. Note that the RCFs have a length ranging from 0.05 to 1.85 mm, which nearly matches the ordinary wood fibers (Table 1, Fig. 3b). However, the type of regenerative solution significantly affects the fiber morphology and structure of the final RCFs. In this study, 4.5 wt% H_2_SO_4_, DI water, 4.5 wt% (NH_4_)_2_SO_4_, and 2.0 wt% NaOH solutions were used to produce RCFs and their corresponding SEM images are shown in Fig. 3c–f. There is a significant reduction in dimension for RCFs in 4.5 wt% H_2_SO_4_ and 2.0 wt% NaOH regenerative solutions (Fig. 3c,f). Interestingly, cellulose nanofiber aggregations with well-proportioned diameters (<100 nm) were harvested by the dissolving-debonding strategy in the 2.0 wt% NaOH solution; their configuration resembles a nanoscale snowflake. This provides a good case for efficiently fabricating regenerative cellulose nanofibers as well as nanocomposites. However, the use of acid and alkaline-regenerative solutions creates disposal challenges and undoubtedly increases the cost of production.

**Figure 4:**
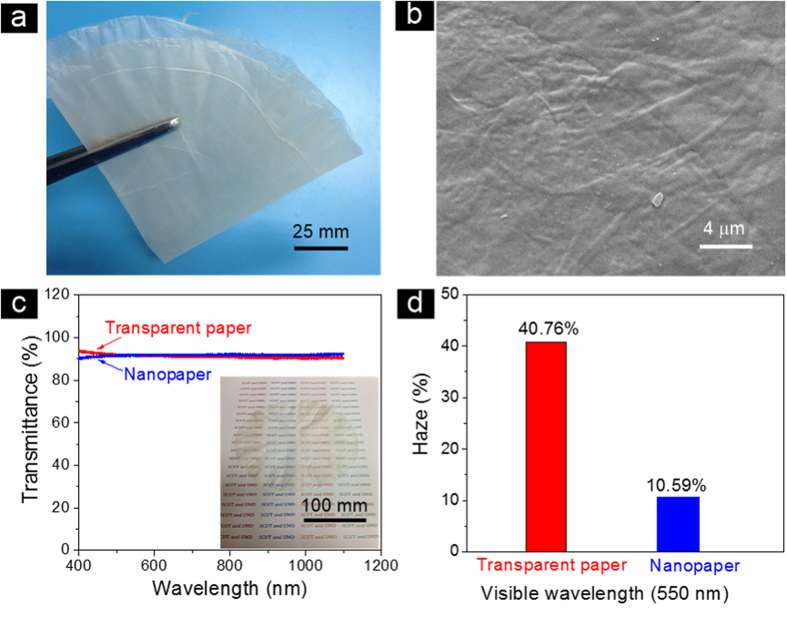
Characterization of the as-prepared paper. (**a**) Digital image of our freestanding, foldable and flexible transparent paper composed of cellulose fibers regenerated in a DI water solution. (**b**) SEM image of our transparent paper showing the fibrous structure. (**c**) Optical transmittance plot for both transparent paper and nanopaper in the range from 400–1100 nm; the inset in the bottom right corner is a digital image of 60 μm thick transparent paper made from 200 mm diameter RCFs. (**d**) Optical haze of our transparent paper and nanopaper at a wavelength of 550 nm.

After dewatering the wet regenerative cellulose matrix, it appeared translucent and was much easier to handle by hand (Supporting Information Figure S1). RCFs regenerated from the DI water solution were successfully used to prepare flexible and foldable transparent paper with densities ranging from 0.5–1.30 g/cm^3^ (Fig. 4a). There are no obvious pores on the transparent paper’s surface as shown in the SEM image (Fig. 4b). Our RCF transparent paper has a total optical transmittance of 91.5% at a wavelength of 550 nm, which is similar to the transparency of nanopaper (Fig. 4c). This transparent paper also demonstrates an optical haze of 40.8% at a wavelength of 550 nm (Fig. 4d), which is much higher than the haze of nanopaper (15–20%) and polyethylene terephthalate (PET) (≤1.0%)^24^ yet lower than previously reported transparent nanostructured paper (~60.0%). Currently, commercial transparent plastic substrates that suit solar cell devices are scarce, and most of them only serve as transparent substrates for fabricating displays. The transparent paper in this work has enormous potential for solar cell devices and other transparent electronics where both high forward transmittance and large optical haze is required.

## Discussion

The RCFs prepared by our dissolving-debonding strategy surpasses conventional cellulose nanofibers and oxidized wood fibers used for producing transparent paper in terms of both fiber preparation time and fiber slurry filtration time. The RCFs preparation time is about 20 min where dissolving and debonding takes 16 min and 4 min, respectively. However, cellulose nanofiber preparation involves time-consuming pretreatment steps and mechanical separation processes that range from 90 min to more than 42 h (Table 2). Much shorter preparation times and simple fabrication steps lead to less energy-intensive transparent paper production. The filtration time for dewatering the fiber slurry (19.3 μm fiber width) through a 38 μm pore size filter is 10 min. This produces a 60 μm thick transparent paper in an astoundingly short amount of time (i.e. 3–48 times less fabrication time) compared to nanopaper. For instance, the filtration time required for dewatering a cellulose nanofiber slurry (5–20 nm fiber width) ranged from 45 min^25^ to 480 min^26^ when an appropriately sized filter medium (0.1–0.65 μm pore size) was used (Table 2). Note that the fiber dimensions must match the pore size of the filter membranes to maintain acceptable dewatering efficiency and keep the fibers on the membrane surface. By using a larger opening filter medium (10 μm pore size), the filtration time required for dewatering a fiber slurry of cellulose nanofibrils (5–20 nm fiber width) is about 30 min. However, the filtration leads to loss of nanofibers since the pores exceed the fiber’s dimensions^27^. Previously, TEMPO-oxidized cellulose fibers with a 26 μm fiber width were used to fabricate both highly transparent and hazy paper (50 μm thickness) in less than 60 min when a 0.65 μm pore size filter medium was used^4^. Nonporous and glossy surface structures composed of RCFs enable water to rapidly filtrate however, the fiber dimensions are on the microscale which limits optical properties. The total time for fiber preparation and slurry filtration is 30 min, which is a magnitude less than previously reported transparent paper. The efficiency of these fiber preparation and dewatering steps could enable roll-to-roll manufacturing of our transparent paper.

**Figure 5:**
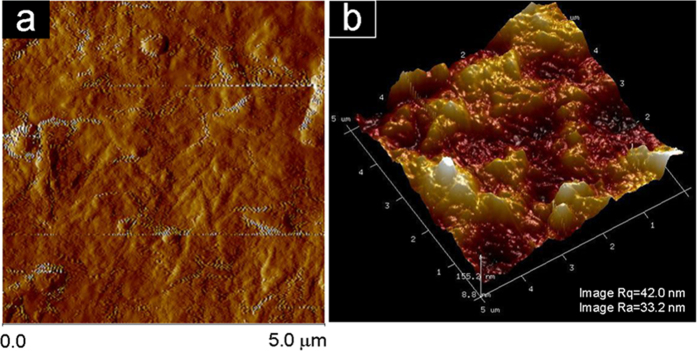
Surface topography of our transparent paper observed by AFM. (**a**) Two-dimensional scanned amplitude and (**b**) 3D scanned height image of the transparent paper.

The surface topography of our transparent paper made of RCFs with an average width and length of 19.3 μm and 0.4 mm respectively was investigated by Atomic Force Microscopy (AFM). The height AFM image of our transparent paper is shown in Fig. 5a where a closed fibrous structure is clearly observed. Additionally, a three-dimensional representation of our transparent paper’s surface is shown in Fig. 5b. The surface RMS (Rq) roughness of the transparent paper was around 42.0 nm, which is much higher than nanopaper (7.7 nm), regenerative cellulose (6.8 nm) and PET (7.0 nm)^15^,^28^.

**Figure 6:**
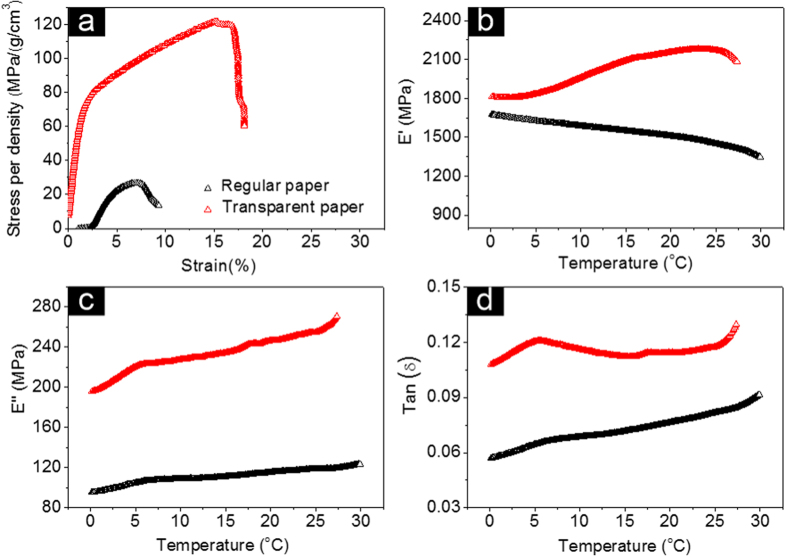
Mechanical properties of our transparent paper and regular paper. (**a**) Specific stress vs. strain curves. (**b**) Storage modulus (E′), (**c**) loss modulus (E″), and (**d**) dissipation factor (Tan(δ)) as a function of temperature.

The transparent paper’s mechanical properties are significant for fabricating various electronic devices. Figure 6a shows the stress-strain curve for both regular paper and transparent paper composed of RCFs from the DI water solution. Our transparent paper shows superior strength compared to regular paper. It was observed that the transparent paper exhibits the highest maximum tensile strength of ~121.69 MPa, a stress of 15.2%, and a toughness of ~8.84 J/M^3^. Note that both the regular paper and our transparent paper are made of microsized cellulose fibers via a similar dehydration process. However, the RCFs have distinctive curved/tangled, nonporous, glossy and homogeneous structures with acceptable elastic properties while the regular wood fibers are loose, hollow, and anisotropic structures that have an abundance of voids (e.g. pits and cell cavities). These structural advantages for the RCFs enable enhanced overlapping between the neighboring fibers and facilitate efficient assembly since tight structures engulf the entire matrix. It was noted that the produced fines (fiber length less than 200 μm) in the debonding process increased the packing density and filled in the voids between neighboring fibers, which undoubtedly increased the packing area and caused the transparent paper to exhibit high mechanical strength and transparency.

Compared to regenerative 4.5 wt% H_2_SO_4_, 4.5 wt% (NH_4_)_2_SO_4_ and 2.0 wt% NaOH solutions, the transparent paper obtained from DI water shows much higher tensile strength (Supporting Information Figure S2). The reason for the decrease in tensile strength stems from the fact that acid or alkaline regenerative solutions are utilized. In this case, the cellulose chains are degraded due to the acid/alkaline conditions which causes the mechanical properties of the RCFs to falter^29^.

To further explore the viscoplastic properties of the transparent paper, dynamic thermomechanic analysis (DMA) was conducted to study the storage modulus (E′), loss modulus (E″) and dissipation factor (Tan(δ)) of our transparent paper and regular paper (Fig. 6b,c). Note that both our transparent paper and regular paper are composed of microsized fibers. The storage modulus (E′) and loss modulus (E″) are indicative of elastic and viscous deformation of materials, respectively. The damping factor (Tan(δ)) refers to the ratio E″/E′ which indicates the material’s viscoelasticity. The storage modulus−temperature curves (Fig. 6b) clearly verifies the outstanding elastic and viscous deformation characteristics of our transparent paper compared to the regular paper. The regular paper demonstrated a storage modulus of 1348.4–1677.2 MPa and a loss modulus of 95.4–123.5 MPa (Fig. 6b,c). However, the transparent paper has a much higher storage modulus of 1803.5–2178.4 MPa and loss modulus of 195.9–269.7 MPa. The dissolving and regeneration process rebuilds the basic structure of the regenerative cellulose fiber, which disorders the original cell wall’s orientational structure. This process reduces the structure’s rigidity but increases the elastic and viscous properties of the RCFs. Nonetheless, the transparent paper demonstrated a twofold increase in damping factor (Fig. 6d) compared to the regular paper, which confirms the transparent paper’s elastic and viscous deformation characteristics. The transparent paper’s superior properties are promising for future stretchable transparent electronic devices.

## Methods

### Dissolution of cellulose fibers

1-ethyl-3-methylimidazolium phosphorous methyl ester (EmimMeOPO_2_H) ionic liquids (ILs) were synthesized using a previously reported method^11^. One part by weight of dimethyl phosphite reacted with 1.15 parts by weight of N-ethylimidazole in THF at 80 °C for 48 h under an argon atmosphere. The resultant was then washed with ether followed by a purification process using dichloromethane and activated, neutral alumina. 0.2 g of cellulose fibers (Sigma-Aldrich (Shanghai) Trading Co., Ltd, China, 99% purity, and medium fiber size) were separately dissolved in 5 ml of ILs at 85 °C through continuous stirring under the anhydrous condition. The process to fully dissolve the cellulose fibers in the ILs took about 16 min.

### Regenerative cellulose film

The cellulose solution was initially cast onto a glass surface. The wet cellulose film was then kept for 30 minutes at room temperature to ensure the basic film’s shape. The cellulose coated glass was then soaked several times in DI water to remove the residual ILs and then dried at room temperature for 72 h under low pressure.

### Preparation of RCFs by the debonding process

300 ml of DI water, 4.5 wt% H_2_SO_4_, 4.5 wt% (NH_4_)_2_SO_4_, and a 2.0 wt% NaOH aqueous solution were utilized to prepare the RCFs. The debonding process is as follows: (1) the regenerative solution was stirred at 5000 rpm to generate strong shear forces and prevent agglomeration; (2) the cellulose solution was then added dropwise into the stirring regenerative solution at a rate of 0.17 ml/min to produce the RCFs. A KajaaniFS300 Fiber Analyzer (FS300, Finland) and an optical microscope (OLYMPUS BX51, Japan) were used to analyze the size distribution and morphologies of the fabricated RCFs.

### Fabrication of transparent paper and nanopaper

The transparent paper was prepared by filtering an RCF slurry onto a glass filter with a nylon fabric membrane (pore size: 38 μm). The obtained wet RCF matrix was repeatedly washed by DI water to eliminate the residual ILs and then dried at 60 °C in a press (BL-6170-B) using 4.0 MPa of pressure. Nanofibrillated cellulose (NFC), with a diameter of ~30 nm, was disintegrated from bleached sulfate softwood pulp according to the method introduced by Zhu *et al.*^11^,^12^. A 0.1 wt% NFC dispersion was poured into a Buchner funnel with a 9 cm PVDF membrane (pore size: 0.65 μm) and vacuum filtrated to remove the water. The obtained wet NFC film was sandwiched between two stacks of regular filter paper to dry under pressure at ambient temperature.

### Characterization

SEM (Philips XL-30, Finland) was used to observe the morphology of RCFs. Both SEM and AFM analysis (Bruker Instruments, Germany) were performed to characterize the surface topography of the transparent paper. The crystal structure of the starting cellulose fibers, regenerative cellulose film, and transparent paper was investigated by an XRD analyzer (Rigaku D/max-III X-ray diffractometer). The tensile strength and dynamic mechanical properties of both the regular paper and transparent paper samples were measured using a universal tensile tester (Instron5565, USA) and a DMA analyzer (DMA 242, German), respectively.

## Additional Information

**How to cite this article**: Chen, J. *et al.* Rapid Dissolving-Debonding Strategy for Optically Transparent Paper Production. *Sci. Rep.* **5**, 17703; doi: 10.1038/srep17703 (2015).

## Supplementary Material

Supplementary Information

## Figures and Tables

**Table 1:** Fiber dimension of the raw pulp fibers and RCFs.

	**Average length (mm)**	**Average width (μm)**	**Average curl index (%)**	**Average kink index (1/m)**	**Fine content (%)**
RCFs	0.4	19.3	52.2	10.9	47.2
Regular pulp fibers	2.4	25.8	18.5	1334.8	8.3

**Table 2:** Comparison of transparent paper prepared using various methods.

	**Diameter**	**Thickness**	**Density**	**Fiber width**	**Pore size of filter**	**Preparation time for fibers**	**Filtration time**	***T***_***550***_	**Refs**
	**(mm)**	**(μm)**	**(g/cm**^3^)	**(nm)**	**(μm)**	**(min)**	**(min)**	**(%)**	
Transparent Paper	200	60	0.5–1.3	19.3 (μm)	38	20 (16 + 4)^a^	10	91.55	This work
Nanopaper	72–200	40–60	0.85–8.73	5–20	0.65	>90^b^	∼45^c^	49.7^d^	15
Transparent Paper	200	50	1.14	26 (μm)	0.65	490	∼60	96	4
Nanopaper	200	—	1.03	5–30	0.65	>490	∼480	90–96	4
Nanopaper	—	60	1.53	15–20	0.1	>2520	180–240	71.6^d^	27
NFC Film	130	120	1.25	5–20	10	—	∼30	—	28

^a^The RCFs took 16 min to dissolve and 4 min to debond.

^b^The cellulose nanofibers took 60 min to undergo enzymatic treatment and 30 min to incubate. The time for homogenization is unknown.

^c^The time for filtrating a 0.2 wt% suspension used to fabricate a 60 μm thick nanopaper with a density of 0.85 g/cm^3^.

^d^Percent transmission at 600 nm wavelength.
