# Supplementary material for: Rapid Dissolving-Debonding Strategy for Optically Transparent Paper Production
Source: Sci Rep. 2015 Dec 11;5:17703. doi: 10.1038/srep17703 (PMC4675992; doi:10.1038/srep17703)
Supplement: Supplementary Information [file srep17703-s1.pdf]

Supplementary Information

Rapid Dissolving-Debonding Strategy for Optically Transparent Paper Production

Jinbo Chen[1¶], Xiaogang Han[2¶], Zhiqiang Fang[2¶], Fan Cheng[1], Bin Zhao[2], Pengbo Lu[1], Jun Li[1], Jiaqi Dai[2], Steven Lacey[2], Raphael Elspas[2], Yuhao Jiang[1], Detao Liu[*1], Liangbing Hu[*2]

1. State Key Laboratory of Pulp and Paper Engineering, South China University of Technology, Guangzhou, Guangdong 510640, People's Republic of China

2. Department of Materials Science and Engineering, University of Maryland, College Park, Maryland 20742, United States

Corresponding authors: L. H. (binghu@umd.edu) and D. L. (dtliu@scut.edu.cn)

J.C., X. H., and Z.F. contributed equally.

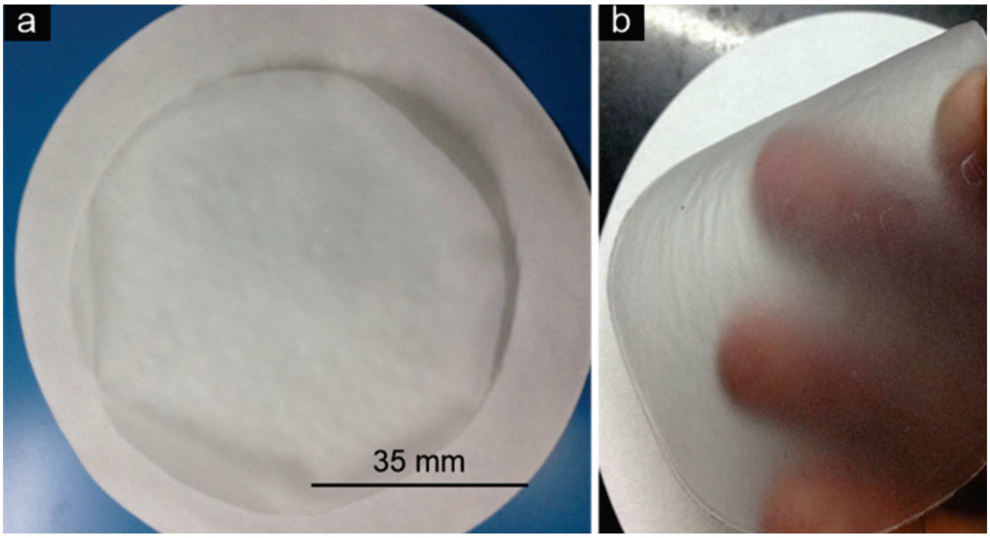

Figure S1. Digital photos of the fibrous mat made of the regenerative cellulose fibers. (a) The as-prepared fibrous mat after dewatering. (b) The free-standing fibrous mat can be handled easily.

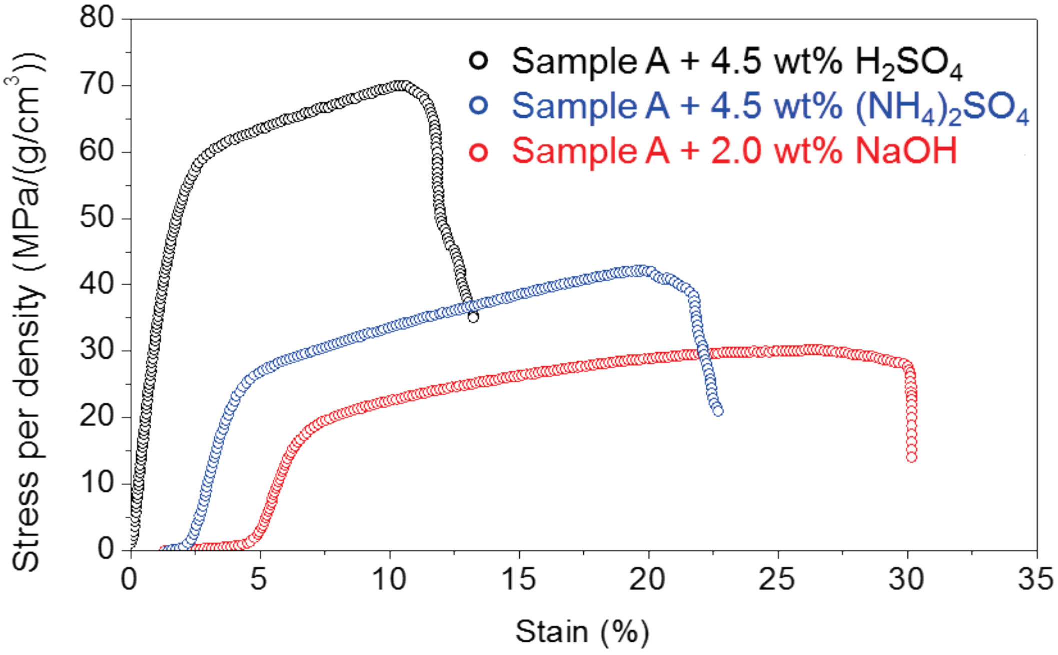

Figure S2. Tensile test results. The comparison of specific stress-strain curves for the transparent papers obtained from the RCFs regenerated in 4.5 wt% H_2SO_4 aqueous solution (Black hollow circle), 4.5 wt% $(NH_4)_2SO_4$ aqueous solution (Blue hollow circle), and 2.0 wt% NaOH aqueous solution (Red hollow circle).
